# A Prospective Study of the Effect of Gastroduodenal Artery Reconstruction on Duodenal Oxygenation and Enzyme Content After Pancreas Transplantation

**DOI:** 10.1007/s00268-023-07149-4

**Published:** 2023-09-12

**Authors:** Juliano C. M. Offerni, Erica Ai Li, Andrew Rasmussen, Wen Y. Xie, Max A. Levine, John Murkin, Vivian C. McAlister, Patrick P. Luke, Alp Sener

**Affiliations:** 1 Department of General Surgery, Division of Urology, University of Manitoba, Winnipeg, MB Canada; 2 Schulich School of Medicine & Dentistry, Western University, London, ON Canada; 3 Division of Urology, Department of Surgery, University of Alberta, Edmonton, AB Canada; 4 Division of Transplantation and Hepatobiliary Surgery, University of Florida, Gainesville, FL USA; 5 Department of Anesthesia & Perioperative Medicine at Schulich School of Medicine & Dentistry, Western University, London, ON Canada; 6 University of Western Ontario, London, ON Canada; 7 Department of General Surgery, London Health Sciences Center, London, ON Canada; 8 Division of Urology, Schulich School of Medicine & Dentistry, London Health Sciences Center, LHSC University Hospital, Western University, C4208, 339 Windermere Road, London, ON N6A 5A5 Canada

## Abstract

**Background:**

Whole pancreas transplantation provides durable glycemic control and can improve survival rate; however, it can carry an increased risk of surgical complications. One devastating complication is a duodenal leak at the site of enteroenteric anastomosis. The gastroduodenal artery (GDA) supplies blood to the donor duodenum and pancreas but is commonly ligated during procurement. Since we have not had expressive changes in pancreatic back table surgical techniques in the recent decades, we hypothesized whether back table GDA reconstruction, improving perfusion of the donor duodenum and head of the pancreas, could lead to fewer surgical complications in simultaneous pancreas-kidney (SPK) transplants.

**Material and Methods:**

Between 2017 and 2021, we evaluated demographic information, postoperative complications, intraoperative donor duodenum, recipient bowel O_2_ tissue saturation, and patient morbidity through the Comprehensive Complication Index (CCI®).

**Results:**

A total of 26 patients were included: 13 underwent GDA reconstruction (GDA-R), and 13 had GDA ligation (GDA-L). There were no pancreatic leaks in the GR group compared to 38% (5/13) in the GDA-L group (*p* = 0.03913). Intraoperative tissue oxygen saturation was higher in the GDA-R group than in the GDA-L (95.18 vs.76.88%, *p* < 0,001). We observed an increase in transfusion rate in GDA-R (*p* < 0.05), which did not result in a higher rate of exploration (*p* = 0.38). CCI® patient morbidity was also significantly lower in the GDA-R group (*s* < 0.05).

**Conclusions:**

This study identified improved intraoperative duodenal tissue oxygen saturation in the GDA-R group with an associated reduction in pancreatic leaks and CCI® morbidity risk. A larger prospective multicenter study comparing the two methods is warranted.

## Introduction

The treatment of diabetes mellitus brings many challenges for both healthcare professionals and patients. From the system perspective, this silent illness demands a large part of the healthcare budget, including treatment, chronic/long-term care, rehabilitation, and hospitalizations of patients affected by this disease. Type 1 diabetes and its sequelae also significantly impact the quantity and quality of life of affected individuals [1].

The worldwide incidence of diabetes has increased in recent decades, with an estimated 463 million peoplebeing affected by this disease in 2019 [2]. Despite recent advances in technology, including continuous glucose monitors and insulin pumps, the number of patients with end-organ dysfunction continues to increase. Although effective, these new treatments are not yet widely available, mainly due to their complexity and cost [3, 4].

Whole-pancreas transplantation continues to play an essential role in treating patients with type 1 diabetes. Patients with type 1 diabetes mellitus and end-stage renal disease (ESRD) may be eligible for simultaneous pancreas and kidney (SPK) transplantation. Whole-pancreas transplant cures patients with type 1 diabetes mellitus by returning them to normoglycemia [5]. This procedure may carry substantial surgical complication profile, including thrombosis and duodenal/pancreatic leakage [6, 7].

The gastroduodenal artery (GDA), which constitutes the main blood supply to the duodenum in 7–30% of patients [8, 9], is typically ligated during the pancreatic pre-transplantation back table to simplify the preparation of the pancreas for implantation. Upon ligation, the duodenum and head of the pancreas become dependent upon the inferior pancreatoduodenal artery (IPDA) from the superior mesenteric artery, which becomes the main contributor of blood supply to the head of the pancreas and duodenum [9, 10]. Therefore, some authors have suggested that hypoperfusion of the donor duodenum and head of the pancreas allograft may lead to perioperative complications of pancreas transplantation, including enteric or pancreatic leaks [8, 11–13]. However, this suggestion was not confirmed in a cohort-matched clinical trial.

As established in the literature, a good blood supply is crucial for adequate tissue oxygen saturation, critical for wound healing and the prevention of anastomotic leaks [14]. GDA reconstruction has been proposed to improve the blood flow to the pancreatic head and donor duodenum, thus mitigating these complications by providing healthier donor tissue to the duodenal bulb, which is required for enteric anastomosis [12, 15].

Despite the theoretical improvement in enteric perfusion by GDA reconstruction, its clinical benefits remain uncommon due to the limited contemporary evidence available on its efficacy in reducing postoperative complications [13]. It was, therefore, the aim of our group to assess the impact of GDA reconstruction on reducing surgical complications in patients undergoing SPK transplantation.

## Methods

### Patients

This study received ethical approval from the institutional review board (REB-103025). We reviewed all adult patients who underwent pancreatic transplantation at our center from February 2017 to August 2021. All recipients were thoroughly assessed and independently cleared to undergo simultaneous pancreas and kidney transplantation by nephrology and transplant surgery teams. Grafts were procured from neurological determination of death (NDD) donors and donors after circulatory death (DCD).

The pancreas was retrieved in a similar fashion by a multi-visceral surgical team during the study period. The transplant surgeon performed back-table graft evaluation and preparation after randomly designating the patient to either GDA reconstruction (GDA-R) or non-reconstruction (GDA-L). Neither patient characteristics nor the surgeon’s preference determined the preference for reconstruction or ligation. Recipient and donor demographics, intraoperative characteristics and postoperative complications were recorded and analyzed.

### Surgical technique

**Fig. 1:**
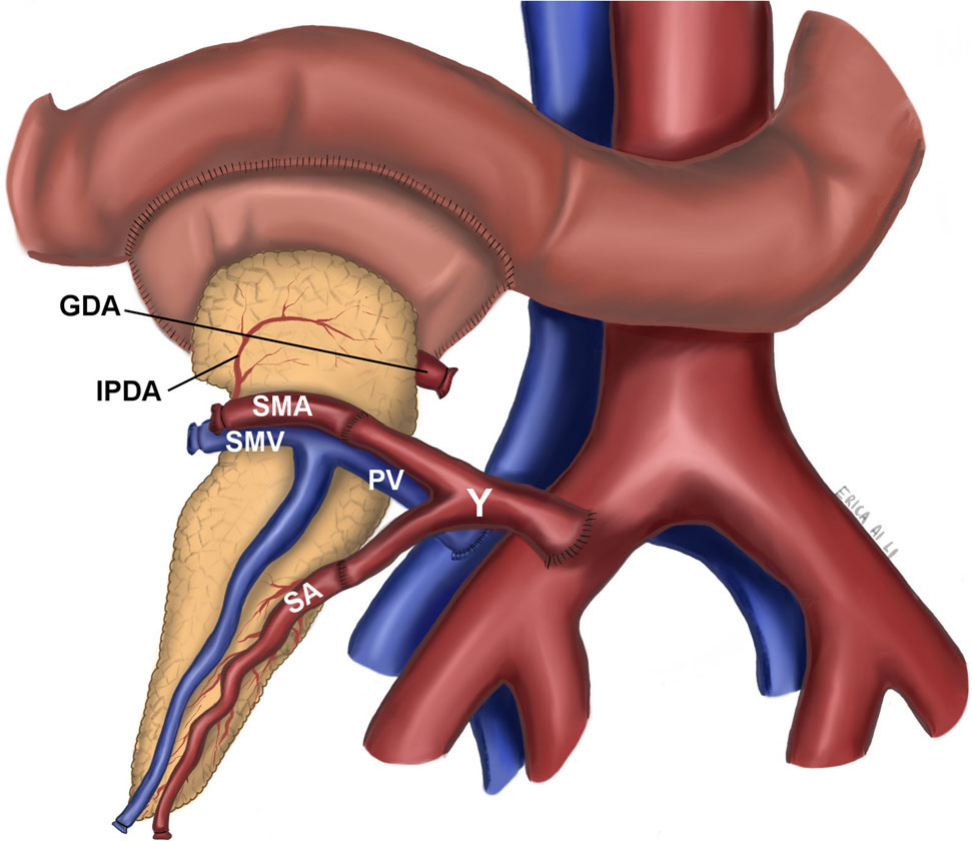
GDA ligation. IPDA, inferior pancreaticoduodenal artery; SMA, superior mesenteric artery; SMV, superior mesenteric vein; PV, portal vein; SA, splenic artery; Y, donor Y graft

One team completed a standard extraperitoneal kidney transplant to the left external iliac artery and vein prior to the pancreas implantation, whereas a second team prepared the pancreas, which was always implanted intraperitoneally in the right iliac vessels. Our center commonly employs a two-surgical team approach to pancreas and kidney transplants. The decision to assign a reconstruction cohort was randomly made before the pancreas back table and not influenced by donor or recipient factors. A Y-graft from the donor iliac artery (common iliac with external and internal branches) is used for vascular reconstruction of all pancreas grafts. In the GDA-L group, the external/internal iliac artery branch of the Y graft is anastomosed end-to-end with the donor superior mesenteric artery (SMA) and end-to-end with the splenic artery. The common iliac artery branch of the Y-graft is then anastomosed to the recipient’s right common or external iliac artery (Fig. 1). The portal vein of the pancreatic allograft is anastomosed to the right common or external iliac vein. Following graft reperfusion, enteric drainage is established by performing a two-layer hand-sew side-to-side enteroenterostomy between the donor duodenum and the proximal loop of the recipient jejunum. The head of the pancreas is aligned with the patient's head, and the tail is carefully positioned behind the bladder to keep it straight.

**Fig. 2:**
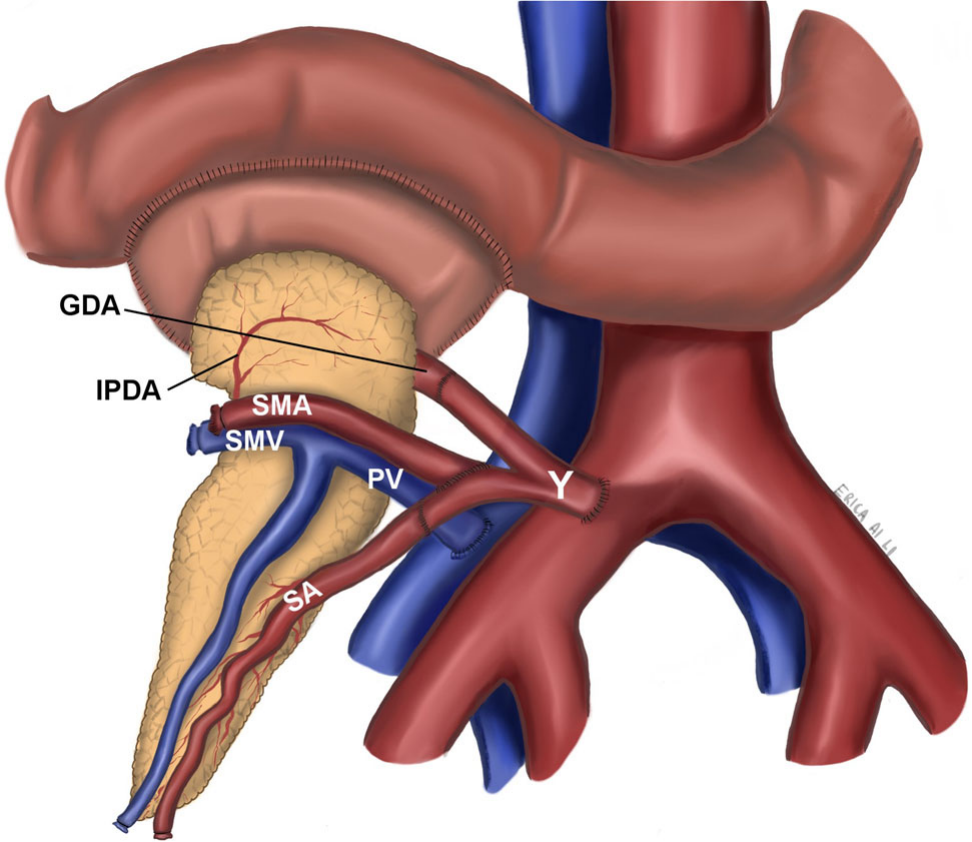
GDA reconstruction. GDA, gastroduodenal artery; IPDA, inferior pancreaticoduodenal artery; SMA, superior mesenteric artery; SMV, superior mesenteric vein; PV, portal vein; SA, splenic artery; Y, donor Y graft

In the GDA-R group, the Y-graft is positioned and carefully evaluated the calibre of the GDA, SMA, and splenic artery before the anastomosis (Fig. 2). One end (internal or external iliac) of the Y-graft is anastomosed end-to-end with the splenic and end-to-side with the SMA artery. The remaining end of the Y-graft is anastomosed end-to-end with the GDA. The portal vein of the pancreatic allograft is anastomosed to the right common or external iliac vein for systemic drainage. Enteric drainage is established by anastomosing the donor duodenal loop side-to-side with the recipient proximal loop jejunum in a two-layer hand-sew manner. The pancreas position afterwards is the same in both groups.

After reperfusion, a device using near-infrared time-resolved spectroscopy (tNIRS) was used intraoperatively to determine tissue oxygen saturation [16]. The tNIRS probe (2 × 1 cm) was positioned in close contact with the tissue to obtain measurements. Five separate measurements were taken from each side of the enteric anastomosis (donor duodenum and recipient jejunum) to ensure saturation variability in different areas of the freshly constructed anastomosis was obtained.

We routinely left surgical drains next to the pancreas and the kidney grafts. We measured amylase or lipase in the pancreatic drain on days 3, 7, 10, 15 and or until the amylase or lipase level reached the serum level, which was used as a reference for drain removal and to define biochemical leak or pancreatic fistula grade B with a persistent drain left in place or percutaneously repositioned after three weeks or grade C if a surgical approach was deemed [17]. The patients were followed postoperatively by a multidisciplinary team of surgeons and nephrologists.

**Fig. 3:**
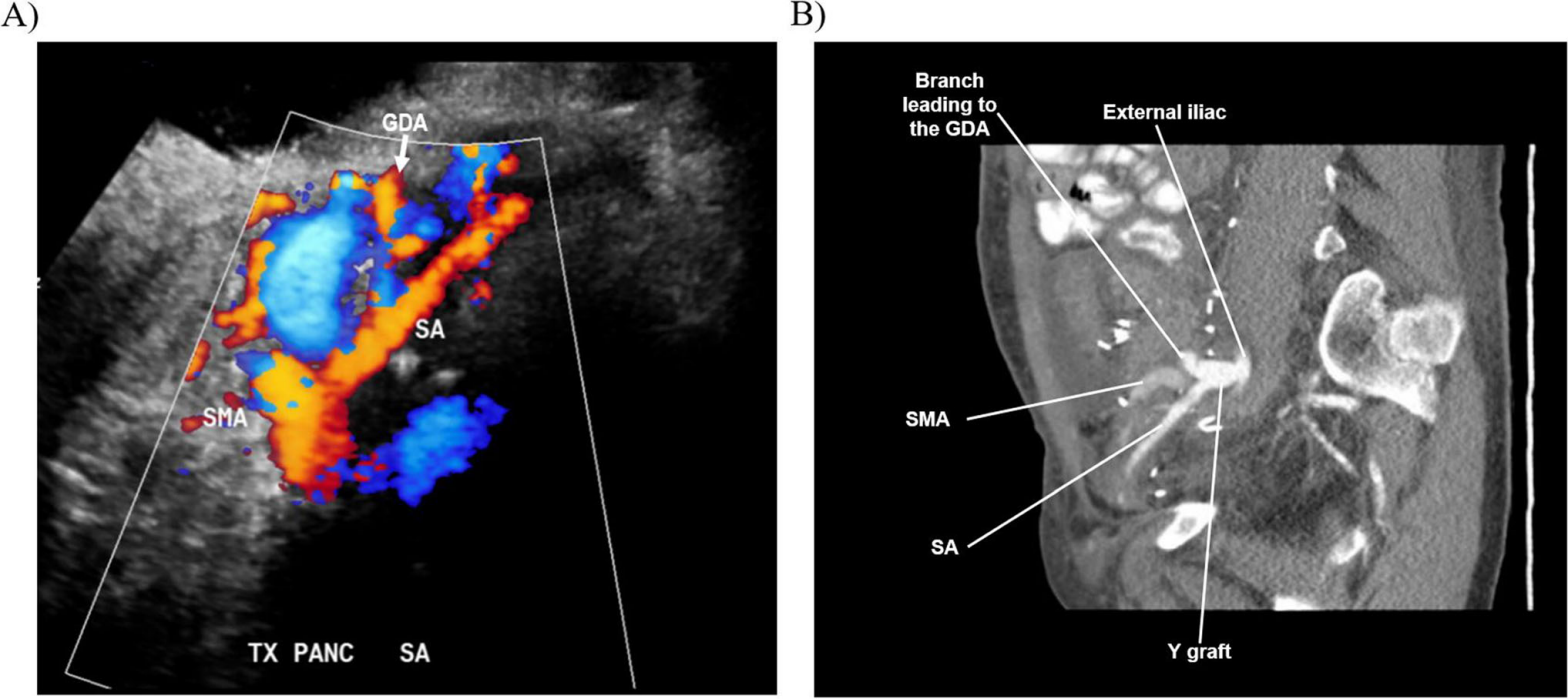
Doppler ultrasound **A** and CT pelvis **B** demonstrating the patent gastroduodenal artery and its communications in the transplant. Both images were taken in the sagittal plane for the same pancreas transplant

Blood sugar levels were measured every 15 min for the first two hours after reperfusion and then every four h for 24 h. Doppler ultrasound was performed routinely on the first postoperative or if sugar did not drop as expected or if it rose after the immediate postoperative period. A doppler ultrasound, as well as CT pelvis, demonstrating the patent gastroduodenal artery and its communications in the transplant are provided in Fig. 3A and B.

### Immunosuppression, anticoagulation, and infection prophylaxis

Our standard immunosuppression induction protocol for SPK transplants included methylprednisone (250 mg IV) and rabbit antithymocyte globulin (rATG) at a dose of 1.5 mg/kg/day for a cumulative target dose of 5–6 mg/kg. We did routinely anticoagulate SPK recipients using a modified low-dose heparin protocol, as previously published by our group [18]. Following a 3000 IU intravenous bolus in the operating room, we started heparin infusion at a rate of 500 IU/hour once the patient reached the transplant inpatient unit, which was then reduced by 100 IU/hour each day for five days [18]. Warfarin was initially on postoperative day 3 with a target INR of 1.5–2.0. Warfarin therapy was typically maintained for three months unless the patient had a thrombotic event postoperatively, in which case it could be extended at the discretion of the transplant surgeon. Postoperative immunosuppression consists of oral tacrolimus (once-daily dosing to a target level of 5–8 ng/ml), mycophenolic acid, and prednisone. Cefazolin and metronidazole are used perioperatively for infection prophylaxis. Recipients are prophylactically treated with valganciclovir for six months if there is a cytomegalovirus (CMV) mismatch between the donor and recipient. Finally, recipients receive antifungal prophylaxis with fluconazole 100 mg/day for 30 days.

### Post-operative complications

An infectious complication was routinely investigated with a CT-scan to determine the presence of intrabdominal collection. A procedure intervention was defined as opening the wound, need for an interventional radiologist to insert a percutaneous abdominal drain, or returning to the operating room. A pancreatic or enteric leak was documented respectively if a fluid collection contained a concentration of amylase and/or lipase in the drain fluid that was three times higher compared with serum and persisted over three weeks or if any enteric content, requiring open or percutaneous repair or drainage [17]. Bowel complications were documented if bowel perforation, obstruction, or perforation was suspected or if abdominal compartment syndrome occurred. Graft loss was defined as allograft pancreatectomy or a total return to insulin therapy. Thrombosis was routinely investigated using ultrasound Doppler on a postoperative day one or if the blood sugar level increased. Patients were immediately returned to the operating room, and vessel exploration was performed by removing clots if feasible, followed by full heparinization. If the graft was considered non-salvageable, allograft pancreatectomy and bowel reconstruction were performed.

### Statistical Analysis

Statistical analysis was conducted using the R software (version 1.1.463, Boston, USA). Patient demographic data, as well as continuous postoperative data, were summarized as mean ± SD. Unpaired t-tests were conducted for continuous variables, and Fisher’s exact tests were conducted for categorical variables.

The cumulative graft and recipient survival rates were assessed using Kaplan–Meier analysis, and the log-rank test was used to compare the two groups statistically.

The severity of surgical complications was based on the modified Clavien-Dindo adjusted for SPK transplantation over a period of 90 days [19–21]. Moreover, we obtained the Comprehensive Complication Index (CCI) to align with the current literature practice in grading surgical postoperative complications based on the Clavien-Dindo Classification. CCI® is a web-based service which comprehensively reflects the overall impact of surgical complications on a scale from 0 to 100. [22]

Statistical significance was set at *P* < 0.05.

## Results

Between February 2017 and August 2021, we performed 26 pancreas transplants, including one pancreas after kidney transplantation, one pancreas alone, one repeat simultaneous kidney and pancreas transplantation, and 23 de novo simultaneous kidney and pancreas transplants. The groups were balanced, with 13 patients in each group. All patients experienced normoglycemia within the first 48 h after pancreatic reperfusion.

The mean follow-up period post-transplant was 923 days (56–1711 days). Early graft failure (first 90 days) occurred in 15.4% (2/13) of patients in the GDA-L group, and no early graft loss occurred in the GDA-R group. Late graft failure (> 90 days) occurred in one patient in the GDA-R group (acute rejection after one year) and one patient in the GDA-L group (patient had severe acute rejection five years after transplantation). The all-cause mortality rate in this study was 3.8% (1/26).

**Fig. 4:**
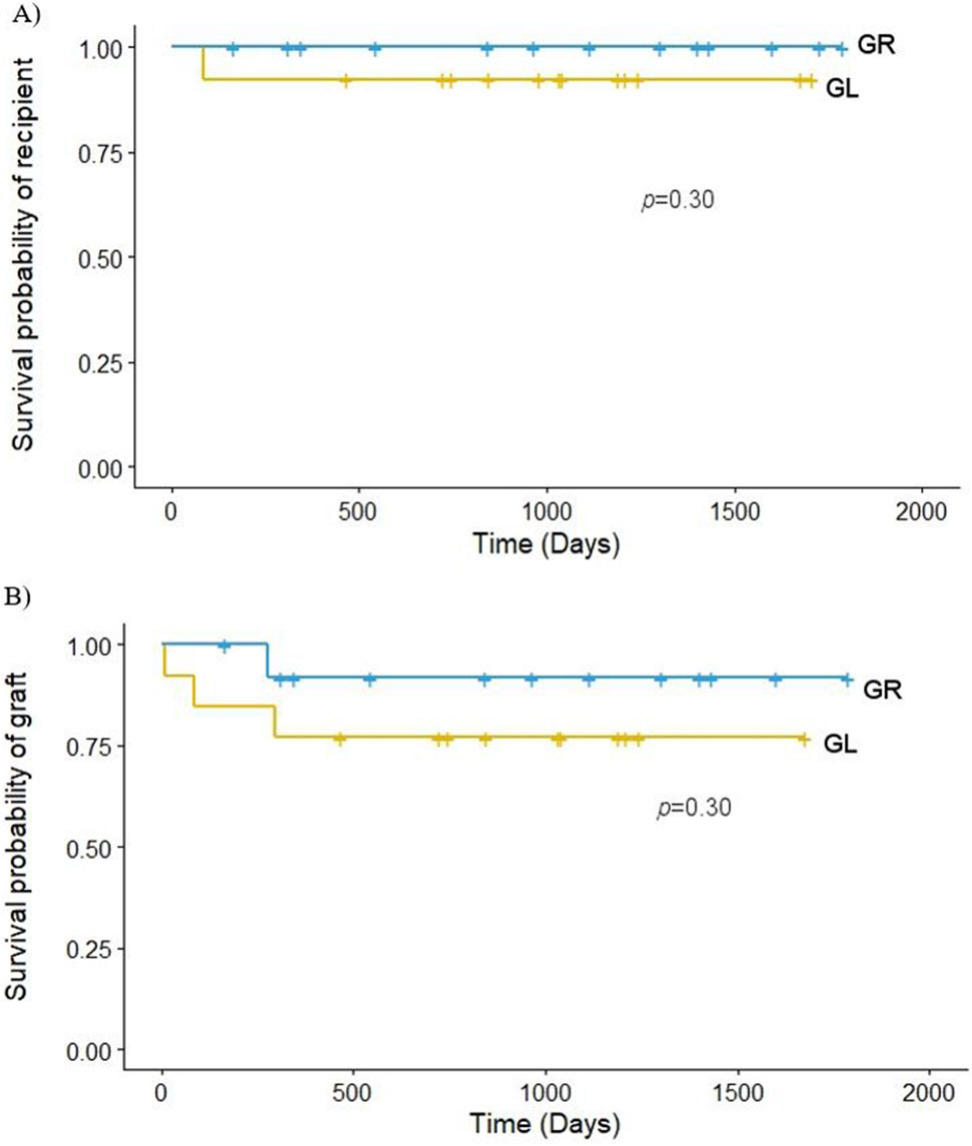
Kaplan–Meier curves for **A** recipient survival and **B** graft survival between GR and GL. Chi-square tests were conducted to compare survival differences

Kaplan–Meier analysis divided by the groups showed a cumulative recipient survival rate of 92.3% in GDA-L and 100% in GDA-R (Fig. 4A) (*p* = 0.30). The cumulative graft survival rate was 76.9% in the GDA-L group and 92.3% in the GDA-R group (Fig. 4B) (*p* = 0.30).

The modified Clavien-Dindo analysis by the group showed that the rates for I, II, IIIA, IIIB, IVA, and IVB severity grades were 20, 0, 20, 40, 10, and 10%, respectively, in the GDA-L (Table 1); and 20, 0, 40%, 40, 0, and 0% in the GDA-R (Table 2). It is essential to point out that if the recipient had more than one complication, we included only the high-grade complication.

**Table 1:** Modified Dindo-Clavien classification for severity of complications in 90 days period in GDA-L

	*GDA-L*									
	Graft Pancreatitis	Infection/Abscess of Pancreatic graft	Focal Necrosis of the Pancreatic Graft	Necrosis of whole Pancreatic Graft	Peripancreatic Graft Fluid Collection	Pancreatic or Duodenal Graft Fistula	Hemorrhage from enteric anastomosis/Intraabdominal hematoma	Graft thrombosis	Death	Total
I	2 (100%,100%)*	0	0	0	0	0	0	0	0	2 (20%)
II	0	0	0	0	0	0	0	0	0	0
IIIA	0	2 (50%,100%)	0	0	0	0	0	0	0	2 (20%)
IIIB	0	2 (50%,50%)	0	0	0	1 (100%,25%)	0	1 (50%, 25%)	0	4 (40%)
IVA	0	0	0	0	0	0	0	1	0	1(10%)
IVB	0	0	0	0	0	0	0	(50%,100%) 0	1 (100%,100%)	1(10%)
*Total*	2 (20%)	4(40%)	0	0	0	1(10%)	0	2 (20%)	1(10%)	10(100%)

*GU* Genitourinary, *I* No complications, *II* Pharmacological treatment, *IIIA* Invasive intervention not requiring general anesthesia, *IIIB* Invasive intervention requiring general anesthesia, *IVA* Graft failure, *IVB* Recipient Death

*The first is percentage for column; the second is for rows

**Table 2:** Modified Dindo–Clavien classification for severity of complications in 90 days period in GDA-R

	*GDA-R*									
	Graft Pancreatitis	Infection/Abscess of Pancreatic graft	Focal Necrosis of the Pancreatic Graft	Necrosis of whole Pancreatic Graft	Peripancreatic Graft Fluid Collection	Pancreatic or Duodenal Graft Fistula	Hemorrhage from enteric anastomosis/Intraabdominal hematoma	Graft thrombosis	Death	Total
I	1 (100%,100%)*	0	0	0	0	0	0	0	0	1 (20%)
II	0	0	0	0	0	0	0	0	0	0
IIIA	0	1 (100,50%)	0	0	1 (100,50%)	0	0	0	0	2 (40%)
IIIB	0	0	0	0	0	0	2 (100%,100%)	0	0	2 (40%)
IVA	0	0	0	0	0	0	0	0	0	0
IVB	0	0	0	0	0	0	0	0	0	0
*Total*	1 (20%)	1 (20%)	0	0	1(20%)	0	2 (40%)	0	0	5(100%)

*GU* Genitourinary; *I* No complications; *II* Pharmacological treatment; *IIIA* Invasive intervention not requiring general anesthesia; *IIIB* Invasive intervention requiring general anesthesia; *IVA* Graft failure; *IVB* Recipient Death

*The first is percentage for column; the second is for rows

There was no IVA or IVB grades in the GDA-R group. The reason for surgical exploration (grade IIIB) in GDA-R was clinically significant hematomas. The two cases that required treatment with minimally invasive intervention (grade IIIA, drain insertion by interventional radiology) turned out to be one infected small hematoma and a small collection, which was neither confirmed to be a pancreatic leak nor an infected collection. The most frequent surgical complication was pancreatic leaks in GDA-L, depicted in Table 3. It was the leading cause of grade IIIB at the modified Clavien-Dindo classification. There was a significant decrease in the rate of pancreatic leaks in the GDA-R group (0/13 patients) compared to the GDA-L cohort (38%, 5/13 patients) (*p* = 0.039). The Comprehensive Complication Index was obtained for each patient, and the mean CCI® was significantly lower in the GDA-R group compared to GDA-L group (*p* = 0.0492) (Table 3).

**Table 3:** Comparing GDA-R to GDA-L for demographic information, intraoperative characteristics, and postoperative complications

	N	GDA-R (Mean ± SD or Incidence (%))	N	GDA-L (Mean ± SD or Incidence (%))	*P*
*Demographic information*					
Recipient Age	13	38.42 ± 5.53	13	42.08 ± 10.6	0.29
Recipient Sex (Proportion Male)	13	5 (38.5%)	13	6 (46.2%)	1.00
Recipient BMI	13	24.41 ± 3.77	13	25.54 ± 4.91	0.52
Recipient Years on Dialysis	10	1.48 ± 0.64	8	1.83 ± 1.31	0.52
Recipient Prior Cardiac Intervention	13	0 (0.0%)	13	1 (7.7%)	1.00
Donor Age	13	29.26 ± 9.56	13	29.80 ± 10.7	0.89
Donor Sex (Proportion Male)	13	7 (53.8%)	13	11 (84.6%)	0.20
Donor BMI	13	25.55 ± 4.13	13	23.83 ± 6.08	0.41
Donor Height	13	173.46 ± 12.4	13	170.85 ± 18.6	0.68
Donor Serum Creatinine	11	67.09 ± 21.0	12	91.67 ± 87.0	0.36
Donor Pancreas Cold Ischemia Time (min)	13	547.00 ± 153	13	607.75 ± 170	0.36
Incidence of DCD donor type	13	6 (46.2%)	13	2 (15.4%)	0.07
Incidence of NDD donor type	13	7 (53.8%)	13	11 (84.6%)	0.07
*Intraoperative Characteristics*					
Procedure Time (min)	13	295.54 ± 32.3	13	274.31 ± 77.7	0.37
Donor Pancreas Warm Ischemia Time (min)	13	33.08 ± 6.51	12	42.58 ± 32.9	0.32
Incidence of Intraoperative Blood Transfusion	13	1 (7.7%)	13	4 (30.8%)	0.32
*Postoperative Complications*					
Peak Drain Amylase D7 (U/L)	9	213.00 ± 236	10	330.60 ± 447	0.49
Peak Drain Amylase D10 (U/L)	9	169.44 ± 157	6	1779.50 ± 2347	0.0567
Peak Drain Lipase D7 (U/L)	5	350.60 ± 247	8	171.75 ± 104	0.0929
Peak Drain Lipase D10 (U/L)	4	168.25 ± 153	4	222.75 ± 209	0.69
Peak Serum Lipase (U/L)	13	197.31 ± 149	11	155.70 ± 133	0.48
Peak Serum Amylase (U/L)	9	300.56 ± 340	10	293.90 ± 249	0.96
Length of hospital stay (post-op)	13	12.08 ± 6.01	13	13.85 ± 7.43	0.51
O2 Saturation Recipient Jejunum	12	95.18 ± 4.31	13	92.03 ± 5.37	0.12
Duodenal Leak	13	0 (0.0%)	13	1 (7.7%)	1.00
Thrombotic Complications	13	0 (0.0%)	13	2 (15.4%)	0.48
Post-Operative Need for Transfusion	11	7 (63.6%)	12	5 (41.7%)	0.41
Overall Incidence of Infection Complication	13	3 (23.1%)	13	5 (38.5%)	0.67
Intra-abdominal Abscess	13	1 (7.7%)	13	2 (15.4%)	1.00
GU Infection	13	1 (7.7%)	13	0 (0.0%)	1.00
Systemic infection	13	2 (15.4%)	13	2 (15.4%)	1.00
Infectious complication requiring procedural intervention	13	2 (15.4%)	13	2 (15.4%)	1.00
Bowel Complications	13	1 (7.7%)	13	0 (0.0%)	1.00
Small Bowel Obstructions	13	1 (7.7%)	13	0 (0.0%)	1.00
Colonic Perforation	12	0 (0.0%)	13	0 (0.0%)	1.00
Fistula Formation	13	0 (0.0%)	13	0 (0.0%)	1.00
Post-Transplant Laparotomy	13	2 (15.4%)	13	5 (38.5%)	0.38
Graft Loss	13	1 (7.7%)	13	2 (15.4%)	1.00
Recipient Death	13	0 (0.0%)	13	1 (7.7%)	1.00
*Statistically Significant Findings*					
O2 Saturation Donor Duodenum	13	93.60 ± 2.92	13	76.88 ± 17.0	< 0.01 (0.0019)
Pancreatic Leak	13	0 (0.0%)	13	5 (38.5%)	< 0.05 (0.03913)
Mean units of pRBC transfused	7	2.29 ± 1.38	5	7.40 ± 5.50	< 0.05 (0.0374)
Comprehensive Complication Index (CCI®)	13	9.88 ± 14.29	13	22.55 ± 16.80	< 0.05 (0.0492)

Unpaired t-tests were conducted for continuous variables and Fisher’s exact tests were conducted for categorical variables

We observed a significantly higher transfusion rate in the GDA-L group (*p* < 0.05), which did not result in a higher re-exploration rate. The rate of post-transplant exploratory laparotomy was 15.4% and 38.5% in the GDA-R and GDA-L groups, respectively (*p* = 0.38). (Table3).

No thrombotic event was identified in the GDA-R group, and two patients developed thrombosis of the pancreatic graft on the first postoperative day in the GDA-L group, as confirmed by ultrasound. Patients were taken back to the operating room, and a thrombectomy was performed, followed by full anticoagulation; however, one patient had the pancreatic graft removed even after the thrombectomy. Important mention that one patient of the GDA-L group had received a pediatric organ; per se, it is considered a high-risk transplant with an increased incidence of vascular complications. The demographic analyses are presented in Table 3. There were no significant differences between the groups in the intraoperative features analyzed, as depicted in Table 3.

Donor duodenal tissue oxygenation saturation after reperfusion was significantly higher in the GDA-R group. A mean of 93.6% was achieved in the GDA-R group (standard deviation, ± 2.92) compared with 76.8% in the GDA-L group (standard deviation: ± 17.0) (*p* = 0.0044) (Table 3). No other significant differences were observed between the groups related to surgical complications. (Table 3). Additionally, after further stratifying the GDA-R and GDA-L groups by NDD and DCD donors, there were no significant differences in postoperative outcomes by donor type (Table 4).

**Table 4:** Comparison of NDD and DCD for post-operative outcomes

	N	NDD Incidence (%)	N	DCD Incidence (%)	*P*
*GDA-R*					
Duodenal Leak	7	0 (0%)	6	0 (0%)	1.00
Pancreatic Leak	7	0 (0%)	6	0 (0%)	1.00
Thrombotic complication	7	0 (0%)	6	0 (0%)	1.00
Graft loss	7	0 (0%)	6	1 (16.7%)	0.46
O2 saturation of the donor duodenum	7	93.43 ± 2.93	6	93.80 ± 3.16	0.83
*GDA-L*					
Duodenal Leak	11	1 (9.1%)	2	0 (0%)	1.00
Pancreatic Leak	11	5 (45.5%)	2	0 (0%)	0.49
Thrombotic complication	11	2 (18.2%)	2	0 (0%)	1.00
Graft loss	11	3 (27.3%)	2	0 (0%)	1.00
O2 saturation of the donor duodenum	11	79.11 ± 15.74	2	64.6 ± 25.46	0.29

## Discussion

Humar et al. showed that 6.5% of pancreatic transplant patients develop a pancreatic leak, a life-threatening complication [23]. Other authors showed an association between relaparotomy and a worse graft survival rate over five years [7]. Duodenal and pancreatic leaks have been attributed to technical factors, ischemia–reperfusion damage, pancreatitis, and the lack of a good blood supply to the head of the pancreas and donor duodenum.

Our study suggests that the adoption of back-table arterial reconstruction of the GDA to improve perfusion of the donor duodenum and pancreatic head could lead to a significant positive impact on pancreatic transplant outcomes. Previous work demonstrates similar benefits for GDA reconstruction, such as reducing pancreatitis, leakage, thrombosis, or bleeding [8, 12, 15]. Increased perfusion to the pancreas head and duodenum has been proposed to mitigate ischemia–reperfusion injury and subsequently improve postoperative outcomes [8, 15].

Diverse factors, including ischemia–reperfusion injury, donor factors, and technical features, can cause posttransplant duodenal and pancreatic leaks. We adopted the recommendations of the last ISGPS update to address issues associated with pancreatic complications. Singh et al. [23] reported that pancreatitis and pancreatic leak, including duodenal stump leak, were major undesirable risks for the development of the peri-graft collection [24]. Significant peri-pancreatic collection can lead to a low pancreatic graft survival rate and a high incidence of associated infections [24]. We found a significant reduction in pancreatic graft leaks in patients who underwent pancreas transplantation in the GDA-R group compared with those who underwent standard ligation of the GDA. Three patients at GDA-L underwent surgical exploration to wash out and drain insertion. Still, it did not result in graft loss, and two patients underwent a percutaneous drain insertion by interventional radiology with no additional treatment. The treatment of pancreatic leak increases the hospital's length of stay, encumbers the health care system, and definitely, increases the morbidity of an already complex procedure, overstressing even more fragile patients. No statistical difference was found in the rate of re-laparotomy between the groups, but the diagnosis of a pancreatic leak was higher in the GDA-L group. The absence of pancreatic leaks in the GDA-R group might be explained by the better blood flow achieved with the reconstruction mitigating the impact of procurement and ischemia–reperfusion injury in the pancreatic graft, decreasing the incidence of pancreatic leaks and pancreatitis [25].

For the first time, we used a clinical tissue oximeter using tNIR time-resolved spectroscopy to document the superior tissue saturation levels in the donor duodenum in the GDA-R group compared to the GDA-L group. In fact, the donor duodenum's tissue oxygenation was not different compared to the oxygenation noted in the recipient jejunum in the majority of patients in the GDA-R group. Although we did not assess blood flow, we confirmed that GDA-R improves perfusion and re-establishes normal oxygenation levels into the tissue, preventing duodenal leak or pancreatic leak, such a devastating complication. Existing literature has shown that a colonic oxygen saturation level below 90%, measured through tissue oximetry, is predictive of an anastomotic leak [14]. A systematic review on tissue oxygenation and anastomotic healing also demonstrated higher leakage rates when oxygenation was poor after colonic anastomosis [25]. Thus, this study provides further evidence to support the link between tissue oxygenation and the risk of anastomotic leakage.

This study had some significant limitations. The small sample size tempers our findings from a single center in our study. In addition, although data on tissue saturation were prospectively collected, complications and graft survival data were retrospectively collected. Despite these limitations, our results support future prospective, multicenter, and randomized studies.

## Conclusions

Reconstruction of the GDA resulted in better perfusion of the donor duodenum, as indicated by tissue perfusion spectroscopy. This may, in turn, reduce the incidence of pancreatic or duodenal leaks, preventing the need for further imaging studies, the need for procedural intervention, or allograft pancreatectomy secondary to an untreatable fistula. The rate of patient morbidity, calculated using the CCI®, was also significantly lower in the GDA reconstruction group. GDA reconstruction was also linked to fewer pRBCs transfused units, reducing the risk of transfusion-associated compilations. A randomized, prospective, multicenter study is warranted to address this question definitively.

## Abbreviations

GDA: Gastroduodenal artery
SPK: Simultaneous pancreas-kidney
IPDA: Inferior pancreaticoduodenal artery
tNIRS: Tissue near-infrared spectroscopy
SMA: Superior mesenteric artery
ATG: Anti-thymocyte globulin
CMV: Cytomegalovirus
ISGPS: International Study Group of pancreatic surgery
DCD: Donor after cardiac death
GDA-L: Gastroduodenal artery ligated group
GDA-R: Gastroduodenal artery reconstruction group
CCI®: Comprehensive Complication Index
